# Efficacy of Two Common Methods of Application of Residual Insecticide for Controlling the Asian Tiger Mosquito, *Aedes albopictus* (Skuse), in Urban Areas

**DOI:** 10.1371/journal.pone.0134831

**Published:** 2015-08-06

**Authors:** Lorenzo Marini, Alberto Baseggio, Andrea Drago, Simone Martini, Paolo Manella, Roberto Romi, Luca Mazzon

**Affiliations:** 1 Department of Agronomy, Food, Natural Resources, Animals, & Environment (DAFNAE), University of Padua, Legnaro (PD), Italy; 2 INDIA srl, Arre (PD), Italy; 3 Entostudio snc, Brugine (PD), Italy; 4 ACTIVA srl, Milano, Italy; 5 Department of Infectious, Parasitic and Immuno-mediated Diseases, Vector-Borne Diseases and International Health Unit, Istituto Superiore di Sanitaà (ISS), Roma, Italy; University of Queensland & CSIRO Biosecurity Flagship, AUSTRALIA

## Abstract

After its first introduction in the 1980’s the Asian tiger mosquito, *Aedes albopictus* (Skuse), has spread throughout Southern Europe. *Ae*. *albopictus* is considered an epidemiologically important vector for the transmission of many viral pathogens such as the yellow fever virus, dengue fever and Chikungunya fever, as well as several filarial nematodes such as *Dirofilaria immitis* or *D*. *repens*. It is therefore crucial to develop measures to reduce the risks of disease transmission by controlling the vector populations. The aim of the study was to compare the efficacy of two application techniques (mist vs. stretcher sprayer) and two insecticides (Etox based on the nonester pyrethroid Etofenprox vs. Microsin based on the pyrethroid type II Cypermetrin) in controlling adult tiger mosquito populations in highly populated areas. To test the effect of the two treatments pre- and post-treatment human landing rate counts were conducted for two years. After one day from the treatment we observed a 100% population decrease in mosquito abundance with both application methods and both insecticides. However, seven and 14 days after the application the stretcher sprayer showed larger population reductions than the mist sprayer. No effect of insecticide type after one day and 14 days was found, while Etox caused slightly higher population reduction than Microsin after seven days. Emergency measures to locally reduce the vector populations should adopt adulticide treatments using stretcher sprayers. However, more research is still needed to evaluate the potential negative effects of adulticide applications on non-target organisms.

## Introduction

After its first introduction in the 1980’s the Asian tiger mosquito, *Aedes albopictus* (Skuse), has started to spread throughout Southern Europe [1–3]. Its high ecological plasticity has allowed the species to successfully spread in both tropical and temperate regions and currently the species is reported for more than 20 European countries [4]. In urban areas the tiger mosquito can quickly spread and reach high population densities due to the presence of catch basins in the drain system [5]. Catch basins are reservoir for collecting surface drainage or runoff that often contain stagnant water that favours reproduction and larval development in absence of natural enemies. For many years the main concern regarding the tiger mosquito was only related to its nuisance to humans, but recently outbreaks of Chikungunya pointed to new risks related to its role as vector of severe diseases [6–9]. For instance, recent increase in human dirofilariosis was linked to transmission by *Ae*. *albopictus* [10]. One solution to reduce the risks of disease transmission is to keep the vector populations at low level. Among the control methods, the development of a more effective chemical control of *Ae*. *albopictus* is urgently needed. Currently, there are several strategies to control *Ae*. *albopictus* at both larval and adult stage. The larval control involves the chemical treatment of the catch basins and other breeding sites [11]. While the control of tiger mosquito is most efficiently accomplished at the larval stage, emergency situations may sometimes require the use of adulticides [12,13]. The control against adult mosquitoes is quite expensive and the results are often effective only in the short term. Negative impacts on non-target arthropods are also higher than those related to larval treatment [14]. Adulticides are, however, very important in case of disease outbreaks to suppress quickly the mosquito population and avoid the further spread of the disease [15–18]. In the USA, the large use of Ultra-Low Volume (ULV) technologies has stimulated research and several studies are available that test the effectiveness of different formulations, spraying devices and spraying timing [18–20]. However, in Europe ULV aerial sprays for mosquito control is still forbidden and residual pesticide applications on mosquito resting sites are the only measure available to control adult mosquito populations [21]. However, the efficacy of residual pesticides application is often constrained by the difficulty in achieving sufficiently high coverage of resting surfaces [22–24] and there is little information about the effectiveness of different application methods. Therefore, understanding the influence of application method on the efficacy of the insecticide becomes a crucial step to improve the control of adult mosquitoes.

In this two-year study we evaluated the efficacy of two methods of residual adulticide application to vegetation against *Ae*. *albopictus* that are largely used in Europe. The effectiveness of the control methods was monitored over time testing also two different commercially available adulticides for mosquito control: Microsin (INDIA, Padova, Italy) and Etox (ACTIVA, Milano, Italy). The study provided key information to improve the chemical control efficacy of adult population *Ae*. *albopictus* in European urban areas.

## Materials and Methods

### Description of the study area

This study was conducted during the summers of 2011 and 2012 (June-October). The experimental area was located in the South-west part of Padua (North East Italy), in the green areas of the Department of Public Health “Ai Colli” of Padua (45°23’N, 011°50’E, c. 15 m a.s.l.). Permissions to carry out the experiments within the private garden were acquired at the beginning of the season by the authorities of the same Department. The area was characterized by a mosaic of buildings and courtyards which formed ideal independent experimental parcels. A dense water catchment basin network was present in the area where mosquitoes could actively breed. Vegetation of courtyards included flowering plants and grass lawns, bushes (0.5–2 m), ornamental trees (2–5 m) and mature trees (5 to >20 m in height). Preliminary observations of the larval abundance in the catch basin showed that *Ae*. *albopictus* was the predominant mosquito species followed by *Culex pipiens*. No other species were observed.

### Experimental design

As the study area presented highly homogeneous vegetation (*Prunus laurocerasus*, *Taxus baccata*, *Tilia* spp., *Platanus* spp., *Pinus pinea*, *Magnolia* spp.), the trials were arranged in a completely randomized design. Between summer 2011 and 2012 a total of six trials were repeated with the same techniques and in the same plots. Every trial was a separate insecticide application where mosquito density was monitored before and after the treatment. Each trial included three different treatments (untreated control, residual insecticide application with mist sprayer and residual insecticide application with stretcher sprayer) with three replications (plots) of each treatment. Half of the trials were performed using Microsin (6^th^ July of 2011, 31^st^ August 2011, and 18^th^ July 2012) and half using Etox 20/20 CE (4^th^ August 2011, 22^th^ September 2011, and 22^th^ September 2012). Each plot was around 0.15 ha (49 x 30 m). The six trials can be considered independent replicates due to the relatively long time intervals between the insecticide applications (average: 26 days) compared with pesticide persistence. For Microsin the manufacture reports a persistence of 8–14 days on vegetation and 15–20 days on walls while for Etox no data is provided. To avoid potential carry-over effects of the previous trial, the pesticide application was replicated when the mosquito density in the treated areas approached the density in the control areas indicating no or very low residual activity of the previous application. We tested difference in human landing between treated and control areas one day before the next application and always found no significant difference (mixed model across the six trials in the two years, d.f. = 2, 151, F = 0.541, P = 0.58).

The two residual adulticide application methods compared were a mist sprayer versus a stretcher power sprayer [25]. The Mounted mist sprayer was an Elite 14S-300, Spray Team Machine, Vigarano Mainarda, Italy (Engine: Lombardini LDW702 series Focus Plus, Diesel a 2 cylinders, 17 HP; Pump: Comet APS41, 40 l/min, 3 membranes, maximum pressure 40 bar; N. 3 brass nozzles double supply, adjustable). The declared average droplet size was around 50–100 μm. Vegetation was treated to the point of runoff (“dripping”) up to 12 m. The mounted sprayer (Sprayteam) is a machine used for applications of relatively large areas such as public gardens, camping etc. Compared to the stretcher, this method treats larger areas per time unit but it does not permit to concentrate the application causing strong insecticide drift. The operator speed was 6–8 km h^-1^. The mist sprayer pressure was 8 bar and the insecticide solution was applied at a dose of 0.37 l m^-2^.

The stretcher power sprayer was Tartaruga 300/30 (BioTecnica Servizi, Via del Lavoro Nord, 25 35040 Urbana (PD) (Engine: Honda GX 160 4 stroke engine 5.5hp; Pump: Comet APS41, 40 l min^-1^, 3 membranes, maximum pressure 40 bar, 100 m tub2 10/18, 80 bar; Handgun Turbo 400 Metal). The declared average droplet size was around 150–200 μm. The stretcher sprayer is a device mainly used in urban areas because it allows access to and treatment of small gardens. The operator applied the insecticide to the vegetation to the dripping point up. This technique permits a careful application of the insecticide but it is generally more time consuming than the mounted sprayer. The operator speed was 3–4 km h^-1^. The mist sprayer pressure was 20 bar and the insecticide solution was applied at a dose of 0.063 l m^-2^.

Beside the two application methods, two insecticides were also compared. Microsin adulticide combined the pyrethroids cypermethrin (10%) and tetramethrin (2%) with the synergist piperonyl butoxide (15%). Etox 20/20 CE adulticide combined the nonester pyrethroid etofenprox (20%) and the pyrethroid tetramethrin (3%) with the synergist piperonyl butoxide (15%). Both insecticides were applied at the recommended concentration of active ingredient of 0.5% for Etox e 0.4% for Microsin. Applications occurred when the weather was clear, dry, with little or no wind and always at the same hour (6:30 pm). The insecticide was applied always by the same operators following the manufacture’s instructions. Their use complied with all relevant regulations regarding the application of insecticides in the study area.

### Mosquito monitoring

In each trial, adult mosquitoes were monitored one day before each insecticide treatment and one, seven, and 14 days after the treatment. To evaluate the mosquito population human landing rate counting was conducted in both treated and untreated areas. The landing rate counting was performed before and after the treatments by the same two trained operators. Mosquito landings on both exposed legs and arms of the first operator were counted by the second operator that also collected the mosquitoes using an aspirator. At each visit, three countings were performed for 5 minutes with a pause of approximately three minutes. Countings were performed in the central area of the plot, in three different points with a distance of approximately 10 m. The points were always the same for the entire study. Mosquito monitoring occurred from 3:00 p.m. to 5:30 p.m. and collected insects were brought to the laboratory for identification. No specific permits were required for the collection of adult mosquitoes. This study did not involve endangered or protected species.

### Ethical issues

The present work was scientifically supported by the Istituto Superiore di Sanità (ISS) (http://www.iss.it/). The ISS is a governmental body that represents the National Health Institute of Italy and the technical-scientific arm of the Ministry of Health. Human landing catches, as well as all the clinical trials involving animals or humans in Italy, were performed following ISS operative procedures and scientific protocols previously authorized by the Ministry of Health, according to the guidelines released by the National Ethical Committee (Decree n. 211, 24 June 2003). Informed consent describing the potential risks connected to the human landing collection procedure was obtained from the two operators before commencing the activity.

### Data analysis

To determine the effects of the insecticide application equipment, insecticide type and time after treatment linear mixed models were used. The response variable was the percent population reduction calculated according the formula as follows:

Percent reduction(%)=[1−(C1xT2/T1xC2)]x100,

where C1 is the number of mosquitoes at the control site before treatment (24 h before), C2 is the number of mosquitoes at the control site after the treatment, T1 is the number of mosquitoes at the treated sites before treatment and T2 is the number of mosquitoes at the treated sites after the treatment [26]. Percent reduction was calculated for one, seven and 14 days after the application. The model included insecticide application equipment, insecticide type and time after insecticide treatment and their interactions as fixed effects, while the random effects were plot nested within trial. The random structure accounted for the spatial dependence in the sampling design (i.e. repeated measures). Analyses were run using the nlme package for R [27].

## Results

**Fig 1:**
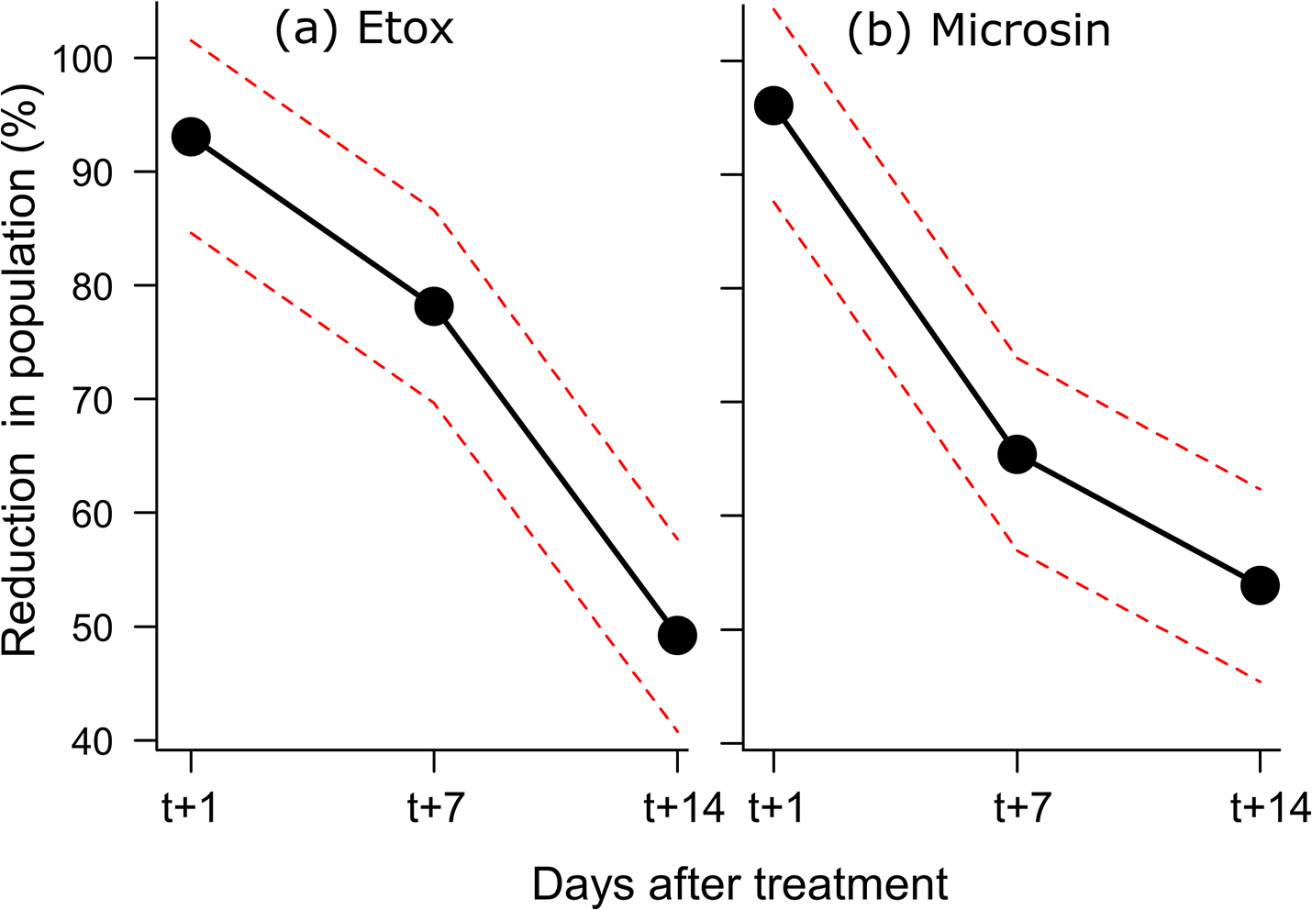
Interaction plot between time after treatment and application method. The dots indicated the average reduction in mosquito population across the six trials.

**Fig 2:**
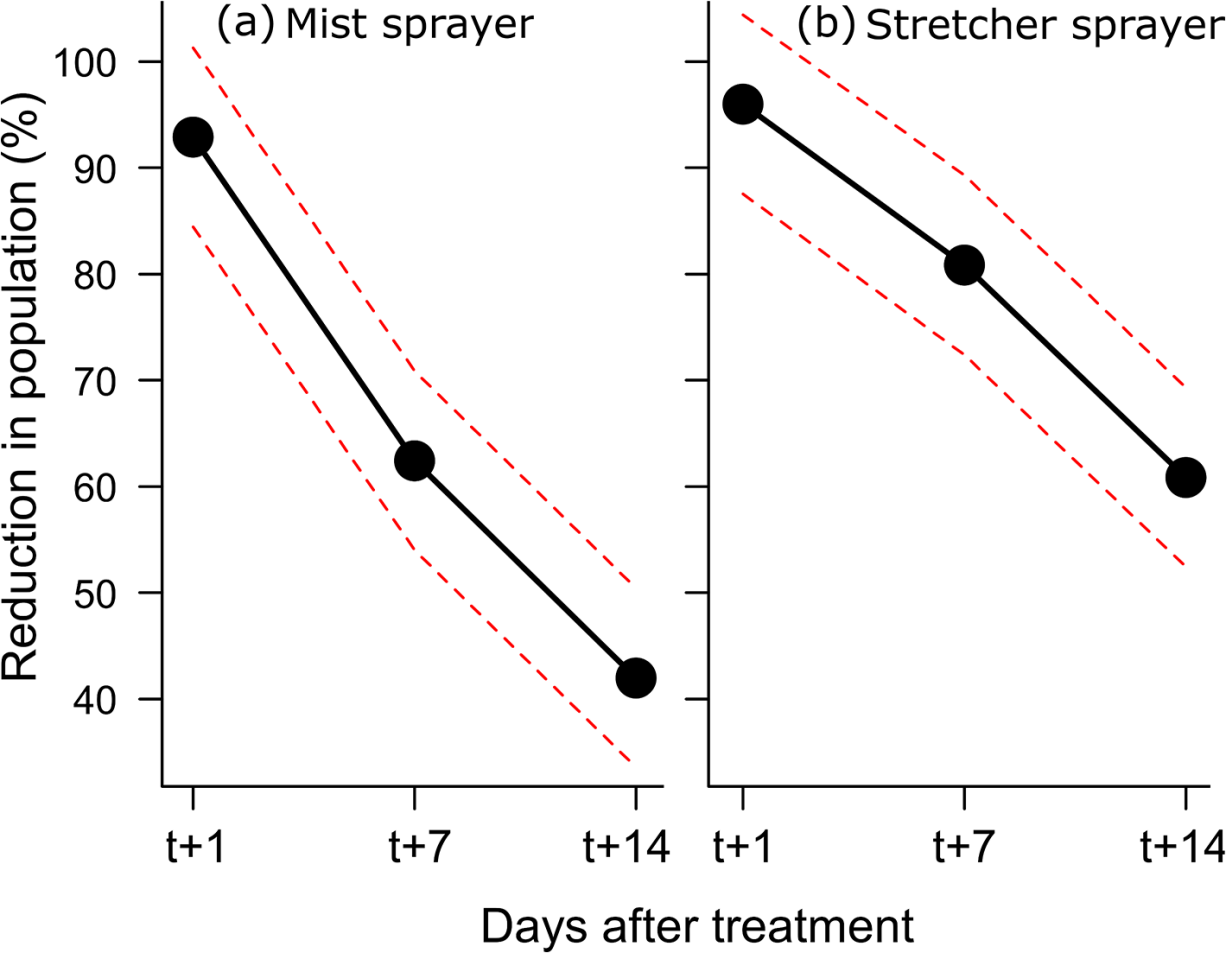
Interaction plot between time after treatment and insecticide type. The dots indicated the average population reduction across the six trials.

The human landing rate counts were conducted for a total of 20 days for a total of 540 counting event and 10,586 *Ae*. *albopictus* collected. As the observations were made only in the afternoon no individual of *Cx*. *pipiens* was collected. In the six trials, *Ae*. *albopictus* was homogeneously present throughout the study area as there was no difference in the number of landings between the different treatments one day before the treatment (all main effects with P>0.05). Time was always significant with the population reduction passing from c. 95% to c. 50% after 14 days (Table 1). The effect of application method was also significant with a higher population reduction of the stretcher compared to the mist sprayer (average across the three times: mist 65% vs. stretcher 78%). The mixed model also indicated a close to significant interaction between time and application method (Table 1). Both application methods had a similar and high reduction after one day while the stretcher had larger reduction after seven and 14 days (Fig 1). After 14 days the Stretcher still had a c. 60% population reduction while the mist sprayer had only c. 40%. Although the main effect of insecticide type was not significant (Table 1), the mixed model indicated a significant interaction between insecticide and time. The interaction indicated that the Microsin had a stronger reduction after seven days than Etox while the reduction after one day and 14 days were not different (Fig 2).

**Table 1:** Results from the linear mixed model testing the effect of insecticide type, application method and time after treatment and their interactions on population reduction (%). The non-significant interactions (P>0.10) were removed using a backward deletion procedure.

	d.f.	F-value	P-value
Application method	1, 14	7.66	0.015
Time	2, 299	63.50	<0.001
Insecticide type	1, 299	0.38	0.534
Application method x Time	2, 299	2.76	0.065
Insecticide type x Time	2, 299	3.16	0.043

## Discussion

The study indicated that one day after the application a very high reduction, close to 100%, of the mosquito populations was obtained by both insecticides and by both application methods. However, after 14 days the population reduction decreased to c. 50%. A significant interaction between the spraying method and time on the population reduction was found, i.e. the effectiveness of the application using the stretcher sprayer lasted more than that of the mist sprayer. Considering the two insecticides the population reduction after seven days was larger for Etox than Microsin while there was no difference just after the treatment. Although adulticide treatments are widely recognized as being less effective than larval control because adult mosquitoes may disperse long distances from the sites where they developed, emergency measures are often required to suppress local adult populations [12,18,28]. However, more research is needed to evaluate the potential negative effects of pesticide applications on non-target organisms [18]. No observations were made in this experiment but negative effects are expected for a wide range of small-bodied arthropods while large-bodied arthropods seem to be less impacted [29]. Our study is a key contribution to identify the most effective application method to control an important vector of severe human diseases.

The efficacy of adulticide application methods under field conditions may be affected by mosquito host-seeking and resting behaviour. In particular, *Ae*. *albopictus* is mainly an exophilic and exophagic mosquito [30] and the protection of some proportion of *Ae*. *albopictus* population resting inside the vegetation may have a negative impact on adulticide efficacy. This can clearly affect the effectiveness of the application of residual insecticides. The effectiveness of insecticides is limited by the effectiveness of application method. If the insecticide does not reach the target then it will not work irrespective of the formulation [18]. The vegetation is a complex filter for the spray, often leading to a reduction in the amount of pesticide available for impact upon a mosquito or its resting sites [31]. The different effectiveness between the stretcher and the mist sprayer can be related to two main causes. First, according to the application procedures the mist sprayer used lower volumes of insecticides per unit area compared to the stretcher sprayer. This implied that larger quantity of residual insecticide can be found on the vegetation for the latter. Second, the penetrative force of the insecticide flow applied with the stretcher sprayer is higher and the insecticide probably reached deeper parts of the vegetation. As pyrethroids can also act as barrier due to their irritating effect, if they are not homogenously applied to the vegetation they can be much less effective in controlling adult mosquitoes. In this context, the mist sprayer is expected to produce a less even cover of insecticide on the vegetation due to a larger drift.

Both formulations tested in this study were a combination of a knock-down pyrethroid and a long, photostable, residual one. Both products used the same knock-down pyrethroid (Tetramethrin), while the residual pyrethroid was different: Cypermethrin (for Microsin) and Etofenprox (for Etox). The slightly better control after seven days obtained with Etox is probably related to the greater residuality of Etofenprox compared to Cypermethrin [32]. The difference, however, disappeared after 14 days when the population reductions reached c. 50% for both insecticides.

In conclusion, this study showed that for small-scale control treatments the stretcher sprayer was more effective than the mist sprayer in controlling adult mosquitoes while the difference between a formulation based on an α-cyano pyrethroid (Cypermethrin) and one based on a nonester pyrethroid (Etofenprox) was less evident. As *Ae*. *albopictus* is considered an epidemiologically important vector for the transmission of many viral pathogens, such as the yellow fever virus, dengue fever and Chikungunya fever, as well as several filarial nematodes such as *Dirofilaria immitis* and *D*. *repens* we need to develop more effective control strategies to suppress vector populations. The implications of our study for adult mosquito management are that small-scale treatments of private gardens should be performed using the stretcher sprayer. Along with efficacy tests of adult mosquito control techniques, more research should be devoted to the potential negative effects of residual insecticides on non-target organisms. This area of research has still received little attention, although the use of residual pyrethroids is a widespread strategy to control adult mosquitos across several European countries.

## Supporting Information

S1 Data Dataset used in the statistical analyses. (TXT)
